# Effect of Contrast Media Type on Renal Function in Percutaneous Coronary Intervention (PCI): A Propensity Score-Matched Analysis Considering Contrast Media Volume and Preoperative Renal Function

**DOI:** 10.7759/cureus.87261

**Published:** 2025-07-04

**Authors:** Tomokazu Takeuchi, Mashio Koike, Yuko Seki, Yasuhiro Fukushima, Ayako Taketomi-Takahashi, Masashi Ando, Noriaki Takama, Takayuki Suto, Yoshito Tsushima

**Affiliations:** 1 Radiology, Gunma University Hospital, Maebashi, JPN; 2 Radiological Technology, Gunma Prefectural College of Health Sciences, Maebashi, JPN; 3 Applied Medical Imaging, Gunma University Graduate School of Medicine, Maebashi, JPN; 4 Diagnostic Radiology and Nuclear Medicine, Gunma University Graduate School of Medicine, Maebashi, JPN; 5 Cardiovascular Medicine, Gunma University Graduate School of Medicine, Maebashi, JPN

**Keywords:** estimated glomerular filtration rate, low-osmotic contrast media, percutaneous coronary intervention, post-contrast acute kidney injury, renal function

## Abstract

Objective

To compare the effects of two low-osmolar contrast media (LOCM), iohexol and iomeprol, on renal function in patients undergoing percutaneous coronary intervention (PCI), as measured by estimated glomerular filtration rate (eGFR).

Methods

This retrospective study included 180 patients who underwent PCI between January 2021 and December 2022. After propensity score matching based on age, sex, diabetes mellitus, pre-PCI eGFR, contrast media volume, and left ventricular ejection fraction, 88 patients who received either iohexol or iomeprol (44 each) were analyzed. Changes in eGFR at 24 hours and more than 72 hours post-PCI were evaluated.

Results

There were no differences between the two groups in age [median 71.5 (range 33-86) vs. 73 (range 50-83) years], gender [35 (79.5%) vs. 37 (84.1%) male], pre-PCI eGFR [64.9 (6.8-112.2) vs. 61.3 (8.5-105.2) ml/min/1.73m²], or amount of LOCM used [108 (42-217) vs. 109 (52-222) ml] between the iohexol and iomeprol groups, respectively. No significant differences in absolute or relative changes in eGFR were observed between the two groups at either time point. At 24 hours, the mean relative change in eGFR was 1.6% (-2.5 to 5.8) in the iohexol group and 1.1% (-2.8 to 4.9) in the iomeprol group. At more than 72 hours, relative changes were -2.1% (-5.9 to 1.7) and -0.3% (-4.4 to 3.8), respectively. No cases of post-contrast acute kidney injury were observed.

Conclusion

Changes in renal function after PCI did not differ significantly between iohexol and iomeprol, despite differences in osmolality and viscosity.

## Introduction

Iodinated contrast media are essential during percutaneous coronary intervention (PCI) [1-4]. Post-contrast acute kidney injury (PC-AKI) is a form of kidney dysfunction that may be induced by contrast media, typically diagnosed within 48 to 72 hours after administration when serum creatinine levels (SCr) increase by ≥50% from baseline or by ≥0.3 mg/dL [5]. The risk of PC-AKI is particularly elevated in patients with preexisting chronic kidney disease, the elderly, individuals with diabetes, and those with heart failure [6-8], and it is associated with prolonged hospital stays and increased mortality following PCI [9,10]. Preventive measures like the use of low-osmotic contrast media (LOCM), minimizing contrast media volume, preoperative fluid replacement, and appropriate post-procedural management may help reduce the risk of renal dysfunction while maintaining PCI efficacy [11-14].

Several studies have evaluated the effects of different LOCM on renal function and the incidence of PC-AKI. One study comparing four LOCM found no significant differences in the incidence of PC-AKI among the agents [15]. While PC-AKI has traditionally been assessed using SCr, estimated glomerular filtration rate (eGFR) may provide a more accurate measure of renal function [8,16]. This is because serum creatinine (SCr) is significantly influenced by factors such as a patient's gender, muscle mass, nutritional status, and age. As a result, impaired renal function can exist even when SCr levels appear "normal". For a more precise assessment, calculated eGFR offers greater accuracy than SCr in predicting true GFR [17]. However, direct comparisons of LOCM effects using eGFR remain limited. Another key factor is viscosity, as high-viscosity LOCM may reduce renal medullary blood flow and increase tubular resistance [18]. To minimize these effects, pre-warming is recommended for LOCM with high viscosity [19].

Two LOCMs are routinely used in the angiography room of our institution. However, no studies have directly compared how their effects on renal function differ in patients receiving intra-arterial LOCM injections. This study aimed to evaluate the relationship between these two LOCMs, which differ in osmolality and viscosity, and eGFR in patients after PCI.

## Materials and methods

Subjects

This retrospective study was conducted in accordance with the Declaration of Helsinki and was approved by the Institutional Ethics Committee of our hospital (No. HS2022-084). The requirement for informed consent was waived; instead, study details were disclosed, and patients who chose not to participate were excluded. All personal information was anonymized, and data analysis was performed after assigning arbitrary identification numbers.

Patients who underwent PCI at our institution between January 1, 2021, and December 31, 2022, were eligible for inclusion. All procedures were performed using one of two LOCM: iohexol (Omnipaque, 350 mgI/mL, GE Healthcare Pharma, Tokyo, Japan) or iomeprol (Iomeron, 350 mgI/mL, Bracco Japan, Tokyo, Japan). The difference between these agents is that iomeprol has lower osmolality and viscosity. Specifically, iomeprol has an osmolality of 0.62 Osmol/kgH₂O, compared to iohexol's 0.83 Osmol/kgH₂O, and a viscosity of 7.0 mPa·s compared to iohexol's 10.6 mPa·s [20]. Patients who underwent an unscheduled emergency procedure or were already on dialysis at the time of PCI were excluded due to the severity of their condition or pre-existing renal impairment. Additionally, patients without left ventricular ejection fraction (LVEF) assessment by echocardiography within four months before PCI were excluded.

Data collection

Data were collected from electronic medical records, including gender, age, height, body weight, presence of renal dialysis and diabetes mellitus, type and volume of LOCMs, and LVEF for patients undergoing PCI. LVEF was primarily measured using the modified Simpson’s method, and the Teichholz method was used when the Simpson’s method was not applicable at echocardiography. Renal function parameters (SCr and eGFR) were assessed before PCI, at 24 hours post-PCI, and more than 72 hours post-PCI, using the closest available blood sampling data.

PC-AKI was defined as an increase in SCr of ≥0.3 mg/dL at 24 hours after PCI compared with the pre-PCI level or an increase of ≥50% from the pre-PCI SCr. The absolute change and relative change in eGFR at 24 hours post-PCI and at more than 72 hours post-PCI were calculated using the pre-PCI eGFR as the reference. The relative change was determined using the following equation (Equation 1).

$\text{Relative change in eGFR}=(\frac{\text{post PCI eGFR}-\text{pre PCI eGFR}}{\text{pre PCI eGFR}})100$　… (1)

Statistical analysis

All statistical analyses were performed using R version 4.3.1 (The R Foundation for Statistical Computing, Vienna, Austria). To minimize the effects of confounding, we performed propensity score matching using the “MatchIt” package. Propensity scores were estimated using a logistic regression model that incorporated potential confounders, including age, sex, presence of diabetes mellitus, pre-PCI eGFR, contrast media volume, and LVEF [21,22]. One-to-one nearest neighbor matching, with a caliper width equal to 0.2 times the standard deviation of the logit of the propensity score, was used to create matched pairs.

Fisher's exact test was used to analyze differences in patient gender, while Welch’s *t*-test was applied to age, contrast media volume, pre-PCI eGFR, and both the absolute and relative changes in renal function at 24 and more than 72 hours post-PCI. A significant level of p < 0.05 was considered statistically significant.

## Results

Baseline characteristics

Of the 350 patients who underwent PCI, 118 emergency cases and 24 dialysis patients were excluded. Another 28 cases were excluded due to the lack of LVEF assessment within four months before PCI. The baseline characteristics before propensity score matching of the remaining 180 patients are summarized in Table 1.

**Table 1:** Baseline characteristics of the study population before propensity score matching, stratified by contrast media type. ^1^n (%); Mean (SD). eGFR = estimated glomerular filtration rate, SCr = serum creatinine, LOCM = low-osmotic contrast media, LVEF = left ventricular ejection fraction.

Characteristics	Iohexol, n = 80^1^	Iomeprol, n = 100^1^
Sex		
Female	16.0 (20.0%)	16.0 (16.0%)
Male	64.0 (80%)	84.0 (84.0%)
Age (years)	68.2 (10.8)	72.6 (8.4)
Height (cm)	164.2 (9.0)	163.0 (8.2)
Body weight (kg)	65.9 (15.0)	66.3 (13.3)
Diabetes mellitus	35.0 (43.8%)	39.0 (39.0%)
eGFR before PCI (ml/min/1.73m^2^)	75.6 (17.2)	54.6 (17.8)
SCr before PCI (mg/dl)	0.8 (0.8)	1.1 (0.5)
Amount of LOCM (ml)	117.6 (42.0)	96.1 (38.0)
LVEF (%)	59.0 (11.9)	57.9 (10.6)

**Figure 1:**
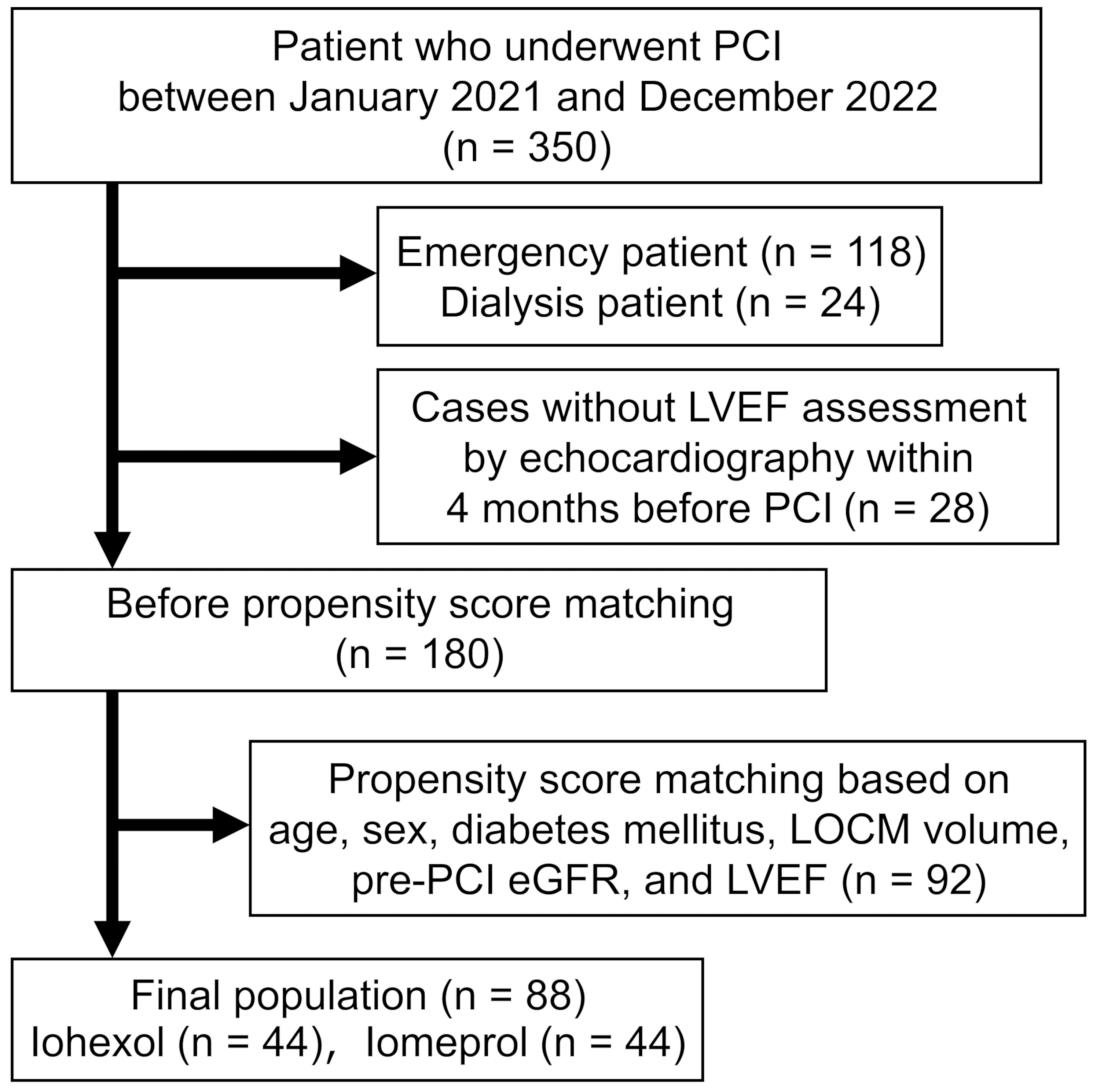
Flowchart showing the study population.

Propensity score matching based on age, sex, presence of diabetes mellitus, contrast media volume, pre-PCI eGFR, and LVEF was applied to the remaining 180 patients. After the subsequent exclusion of 92 patients, a total of 88 patients (44 receiving iohexol and 44 receiving iomeprol) were included in the final analysis (Figure 1).

The baseline characteristics after the propensity score-matched analysis showed no significant differences between patient groups (Table 2).

**Table 2:** Baseline characteristics of the study population after propensity score matching, stratified by contrast media type. ^1^n (%); Mean (SD). eGFR = estimated glomerular filtration rate, SCr = serum creatinine, LOCM = low-osmotic contrast media, LVEF = left ventricular ejection fraction.

Characteristics	Iohexol, n = 44^1^	Iomeprol, n = 44^1^
Sex		
Female	9.0 (20.5%)	7.0 (15.9%)
Male	35.0 (79.5%)	37.0 (84.1%)
Age (years)	70.6 (10.7)	70.8 (8.3)
Height (cm)	162.5 (8.9)	161.7 (7.3)
Body weight (kg)	63.8 (13.4)	64.9 (8.8)
Diabetes mellitus	16.0 (36.4%)	15.0 (34.1%)
eGFR before PCI (ml/min/1.73m^2^)	69.3 (17.7)	66.9 (18.1)
SCr before PCI (mg/dl)	1.0 (1.0)	0.9 (0.6)
Amount of LOCM (ml)	111.4 (41.0)	113.8 (37.4)
LVEF (%)	58.8 (11.9)	58.4 (9.9)

Changes in renal function before and after PCI

**Figure 2:**
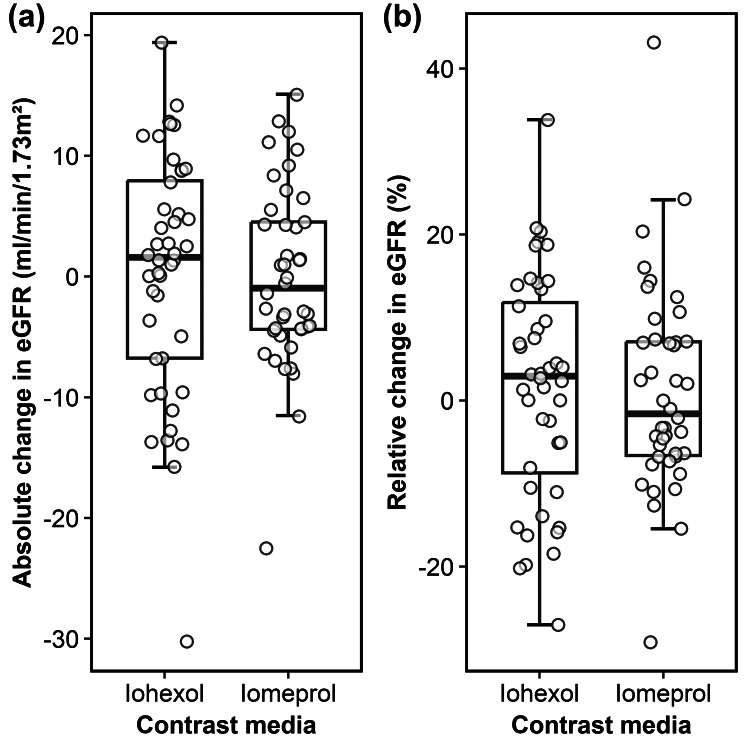
Box plots showing (a) the absolute and (b) relative change in eGFR at 24 hours post-PCI for iohexol and iomeprol. No significant differences were observed between the two LOCM. eGFR = estimated glomerular filtration rate; PCI: percutaneous coronary intervention; LOCM: low-osmolar contrast media.

**Figure 3:**
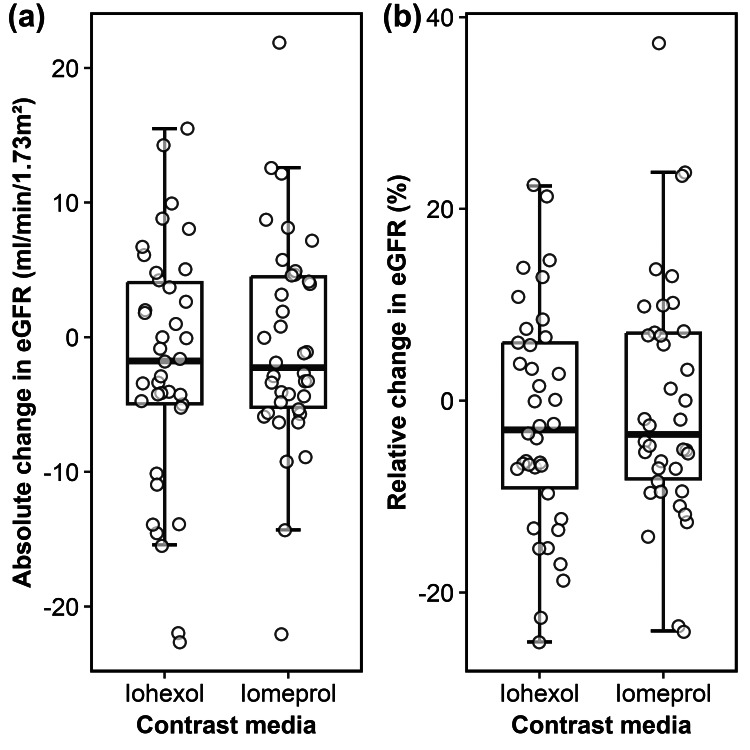
Box plots showing (a) the absolute and (b) relative change in eGFR more than 72 hours post-PCI for iohexol and iomeprol. No significant differences were observed between the two LOCM. eGFR = estimated glomerular filtration rate; PCI: percutaneous coronary intervention; LOCM: low-osmolar contrast media.

The changes in eGFR at both 24 hours post-PCI and more than 72 hours post-PCI in the iohexol and iomeprol groups are illustrated in Figures 2, 3, respectively. At 24 hours post-PCI, the mean absolute change in eGFR was 0.3 mL/min/1.73 m² (95% CI: -2.7 to 3.4) in the iohexol group and 0.0 mL/min/1.73 m² (-2.4 to 2.3) in the iomeprol group, while the mean relative change was 1.6% (-2.5 to 5.8) and 1.1% (-2.8 to 4.9), respectively (Figure 2).

At more than 72 hours post-PCI, the mean absolute change in eGFR was -2.0 mL/min/1.73 m² (-4.9 to 1.0) in the iohexol group and -0.6 mL/min/1.73 m² (-3.2 to 2.0) in the iomeprol group, with corresponding relative changes of -2.1% (-5.9 to 1.7) and -0.3% (-4.4 to 3.8) (Figure 3).

Thus, no statistically significant differences were observed between the two groups at either time point. PC-AKI was assessed based on renal function at 24 hours post-PCI, with no cases observed after propensity score matching of pre-PCI renal function.

## Discussion

No statistically significant differences in the absolute or relative changes in eGFR from pre-PCI to eGFR at 24 hours post-PCI and more than 72 hours post-PCI were observed between the two LOCMs. Regarding the incidence of PC-AKI, no cases were detected in any patients. These findings suggested that renal function after PCI was not significantly different between iohexol and iomeprol.

The two LOCM used in this study differ in osmolality. Prior studies have reported a reduced risk of adverse events―such as major adverse renal events (MARE), major adverse renal and limb events (MARLE), and major adverse renal and cardiac events (MARCE)-with iso-osmolar contrast media (IOCM) due to their lower osmolality compared to LOCM [23]. Our findings indicated that within the LOCM category, these differences in osmolality did not result in significant changes in renal function. These results aligned with previous findings indicating no significant difference in the incidence of PC-AKI between LOCM [15].

In addition to osmolality, viscosity may be another key factor influencing renal function. Previous animal studies have demonstrated that IOCM with high viscosity increased intra-tubular pressure, leading to reductions in glomerular filtration rate and renal blood flow [18,24,25]. The LOCM used in this study has relatively low viscosity compared to IOCM, which may have minimized its impact on renal blood flow. Although no cases of PC-AKI were observed, statistical analysis revealed no significant differences in renal function decline between iohexol and iomeprol, suggesting that the viscosity differences between these two LOCM may not have a clinically meaningful effect on renal function. Based on previous findings [19,26], the lower viscosity of iomeprol may offer advantages in clinical settings. Studies have suggested that lower-viscosity LOCM may reduce injection pressure and may lower hand injection pressure when using small catheters. One study also reported that low-viscosity LOCM can reduce angioplasty balloon deflation time, which may be beneficial in interventional procedures [27]. Given that both agents demonstrated comparable renal safety in this study, the lower viscosity of iomeprol may be a favorable factor when selecting a contrast medium for PCI.

This study had several limitations. First, as a single-center, retrospective study, the findings may have limited generalizability, although the consistent and well-documented medical records used in this study may ensure the reliability of the data. Second, the study included a small number of patients with pre-PCI eGFR<30 mL/min/1.73 m², making it difficult to conclude that those with severe renal dysfunction. Thirdly, renal function assessments at more than 72 hours post-PCI may have been influenced by variability in the timing of blood sampling across patients. Finally, this study did not account for certain patient background factors, such as underlying diseases or diabetic medication use, beyond those specifically investigated. Consideration of additional clinical details, including medical history related to renal function and prior contrast media exposure, may yield different results.

## Conclusions

Several LOCMs are routinely used in the angiography room. However, no studies have directly compared how their effects on renal function differ in patients receiving intra-arterial LOCM injections. In this study, we compared the effects of two LOCMs, iohexol and iomeprol, on renal function. Changes in renal function after PCI did not differ significantly between the two LOCMs, despite differences in osmolality and viscosity. Our findings suggested that the difference between the effects of iohexol and iomeprol on eGFR was limited.
